# Association of Serum Extracellular Vesicle miRNAs with Cognitive Functioning and Quality of Life in Parkinson’s Disease

**DOI:** 10.3390/biom14081000

**Published:** 2024-08-13

**Authors:** Paulina Vaitkienė, Aistė Pranckevičienė, Andrius Radžiūnas, Augustina Mišeikaitė, Giedrė Miniotaitė, Violeta Belickienė, Ovidijus Laucius, Vytenis Deltuva

**Affiliations:** 1 Laboratory of Molecular Neurobiology, Neuroscience Institute, Medical Academy, Lithuanian University of Health Sciences, Eiveniu Str. 4, LT-50009 Kaunas, Lithuania; 2 Health Psychology Department, Faculty of Public Health, Medical Academy, Lithuania University of Health Sciences, Tilžės g. 18, LT-47181 Kaunas, Lithuania; aiste.pranckeviciene@lsmu.lt; 3 Neuroscience Institute, Medical Academy, Lithuanian University of Health Sciences, Eiveniu Str. 4, LT-50009 Kaunas, Lithuania; 4 Department of Neurosurgery, Medical Academy, Lithuanian University of Health Sciences, Kauno Klinikos, Eiveniu Str. 2, LT-50009 Kaunas, Lithuania; 5 Faculty of Medicine, Medical Academy, Lithuanian University of Health Sciences, A. Mickeviciaus g. 9, LT-44307 Kaunas, Lithuania; 6 Neurology Department, Lithuanian University of Health Sciences, Kauno Klinikos, Eiveniu Str. 2, LT-50009 Kaunas, Lithuania

**Keywords:** Parkinson disease (PD), cognitive functioning, blood serum EVs, miR-153, miR-200a, microRNA

## Abstract

The identification of mechanisms associated with Parkinson disease (PD) development in cognitive functioning would be of great usefulness to clarify PD pathogenesis and to develop preventive and therapeutic strategies. In this study, blood serum extracellular vesicle (EV) levels of the candidate microRNAs (small noncoding RNAs that play a role in gene expression regulation):,miR-7, miR-21, miR-153, miR-155, miR-200a and miR-214, have been investigated for association with PD in a group of 93 patients with cognitive parameters, PD symptoms, affected quality of life and some clinical characteristics. MiRNA was extracted from patients’ blood serum EVs, transcribed into cDNA and their expression was evaluated using RT-PCR. The miR-153 and miR-200a showed the most plausible correlations with cognitive functioning parameters such as general intellectual functioning, psychomotor speed, mental flexibility, and nonverbal executive functions. Moreover, lower levels of miR-153 were associated with attention span, working memory and psychomotor speed with learning. Increased levels of miR-200a, miR-7, miR-214, and miR-155 were also linked with neurological functioning, such as bradykinesia, tremor, balance and others. Despite the fact that due to small sample size, our results should be considered as preliminary, our study suggests that miRNA expression in EVs could be associated with symptom severity, cognitive impairment and quality of life in PD.

## 1. Introduction

Parkinson’s disease (PD) is considered the second most common neurodegenerative disorder, with a mean onset age of 60 years [1]. It is characterized by the progressive loss of dopaminergic neurons and the accumulation of α-synuclein, which leads to neuronal dysfunction [2]. The initial diagnosis is often based on clinical motor symptoms, such as bradykinesia, rigidity or tremor; however, other symptoms that are identified as non-motor can also occur frequently and significantly affect the quality of life. Cognitive impairment (CI) is the most prevalent and debilitating nonmotor symptom in PD and the risk factor for the development of dementia [3,4]. The molecular mechanisms underlying cognitive functionality and the increased risk of progression to dementia are not well understood [3,5]. Despite significant recent studies of PD, challenges remain in effectively treating both motor and non-motor symptoms [6]. As the pathology of PD develops, cells undergo various changes, send signals, and share biological information. Identifying biomarkers that can provide useful information about these changes would be valuable for studying disease progression and potential interventions [7].

One promising type of biomarker is miRNA molecules. MiRNA is a small single-stranded non-coding RNA molecule and is an important regulator of gene expression, by binding to the 3′ untranslated regions of messenger RNA (mRNA), resulting in translational repression of the target [8]. MiRNAs can modulate the accumulation of α-synuclein proteins by regulating the expression of proteins that regulate survival of neuronal cells [9]. MiRNAs regulate the amount of mRNA in various organs, including the brain. When miRNA regulation is disturbed, pathological processes of neurodegeneration may occur. In this context, miRNAs could be involved directly and indirectly in the pathogenesis of PD and have a potential therapeutic value [10]. 

The activity of miRNA is not limited to the cells where it is formed [11]. It has been investigated that most cells, including neurons, microglials and astrocytes, secrete EV’s [12]. MiRNAs’ can be packaged into exosomes or microvesicles, infiltrate the extracellular space and travel through biological fluids and provide long lasting effect of disease-related genes [13]. Studies suggest that EV miRNAs can be considered promising biomarkers for PD due their ability to cross the blood–brain barrier and their presence in CSF, peripheral circulation and exosomes [14]. 

More and more interfaces are emerging that miRNAs contribute to cognitive impairment by playing critical roles in neurodevelopment, synaptic plasticity, memory and the regulation of neurodegenerative disease-associated pathological proteins [15,16]. However, to our knowledge, only few studies have previously investigated the roles of miRNAs in cognitive impairment and severity of symptoms in PD [16,17]. We aimed to investigate whether α-synuclein-related serum EV miRNAs (miR-7, miR-153, miR-214, miR-155, miR-21, and miR-200a) are associated with symptom severity, cognitive impairment, and quality of life in PD patients. It is well established that miR-7, miR-153 [18] and miR-214 [19] directly target α-synuclein (SNCA) mRNA, thereby reducing its expression. This regulation is crucial because the overexpression of α-synuclein leads to its aggregation, a hallmark of PD. In contrast, miRNAs such as miR-155 [20], miR-21 [21], and miR-200a [22] do not directly target SNCA mRNA but influence pathways that affect α-synuclein aggregation. Our study aimed to explore the potential of EV miRNAs for clinical use in diagnosing and treating PD, given their significant deregulation in PD models.

## 2. Materials and Methods

### 2.1. Participants/Study Cohort

Data were gathered in two waves. The enrollment to the first study took place between January 2015 and April 2017. Adult patients diagnosed with Parkinson’s disease were recruited in this prospective observational cohort study from Departments of Neurosurgery and Neurology of the Lithuanian University of Health Sciences Hospital, Kaunas, Lithuania. The study inclusion criteria covered established diagnosis of L-DOPA responsive idiopathic Parkinson’s disease, normal brain MRI scan results, MMSE score greater than 24 points, and no active or untreated depression and comorbid psychiatric disorders. The study exclusion criteria were atypical Parkinsonism, diagnosis of dementia or other current or past psychiatric disorders and clinical comorbidities that precluded from the DBS implantation surgery. Preoperative blood serum samples of 19 PD patients who later underwent the DBS implantation and 27 control patients, who remained in conservative treatment, were available for current analysis. 

The second wave took place from June 2020 to July 2021. During this stage all PD patients referred to neurosurgical treatment (Gamma knife stereotactic radiosurgery, GK SRS, Radiofrequency ablation, RFA, or Deep Brain Stimulation, DBS) at the Department of Neurosurgery were invited to participate in the study. The inclusion criteria for DBS neurosurgery were the same as during the first wave. Patients to whom DBS was contraindicated due to clinical comorbidities, older age or decreased cognitive functioning were offered GK SRS or RFA treatment. Preoperative blood serum samples were available for 20 patients who later underwent the DBS implantation, 13 patients who underwent GK SRS and 6 patients who were treated using the RFA, and 10 patients, who remained in the conservative treatment. 

Blood samples were drawn upon hospital admission for neurosurgical cases and during routine neurological visit for conservatively treated cases. Venous blood samples were centrifuged, and serum samples were stored frozen in −80 °C. Neuropsychological assessment was performed by a certified medical psychologist. Medical history, clinical characteristics, and functional status of the study patients were recorded by the study neurologist and neurosurgeon.

The study and its consent procedures were approved by the Ethics Committee for Kaunas region Biomedical Research at the Lithuanian University of Health Sciences, Kaunas, Lithuania (BE-2-3 and BE-2-48). Each patient gave signed informed consent before enrollment in the study.

### 2.2. Psychological Assessment

Several neuropsychological tests were used for more comprehensive assessment of PD patients neuropsychological functioning. Due to involvement of the motor function as well as the declined general state of health, it was not always possible to perform complete psychological assessment. The percent of patients having each assessment is displayed in Supplement Table S1. 

The Trail Making Test (TMT, Parts A and B) was used for assessment of visual attention, psychomotor speed, mental flexibility, and executive functioning [23,24]. The task requires a patient to connect a sequence of 25 targets (numbers 1, 2, 3 etc. in Part A, and alternate between numbers and letters 1, A, 2, B etc. in Part B) on a sheet of paper.

The Rey Auditory Verbal Learning Test (RAVLT) was used for assessment of verbal memory functions [25,26]. The test is based on the list learning paradigm; several aspects of learning process, such as working memory, acquisition of new information, susceptibility to interference, recall and recognition, can be evaluated. The test consists of 15 unrelated nouns (List A) that are read aloud for five trials; each trial is followed by a patient’s free recall. After five trials, a second “interference” list of unrelated words (List B) is given and asked to be recalled. After the interference trial, the patient must again immediately recall nouns from List A. Delayed recall of List A is evaluated after approximately 20 min filed with other cognitive tasks to prevent rehearsal. 

The Verbal Fluency Test was used to evaluate verbal executive functions. This test consists of phonemic and semantic fluency tasks, that were administered according to recommendations of Strauss et al. and adapted for the Lithuanian language [27,28]. During the phonemic fluency task, the patient is asked to produce as many words as possible beginning with a specific letter within a one-minute interval. For the assessment of semantic fluency, patients were asked to produce as many animal names as possible within a one-minute interval.

Digit Span and Digit Symbol Coding subtests from the Lithuanian version of the Wechsler Adult Intelligence Scale-III were used to assess the attention span/working memory and psychomotor speed [29]. Digit Span contains two different tasks. For the Digit Span Forward, the subject’s task is to repeat given sequences of increasing length as they are given. During the Digit Span Backwards, on hearing the sequences, the subject’s task is to repeat them in exactly reversed order. During the Digit Symbol Coding task, the subject must fill in the blank spaces with the symbol paired to the number in the key above. The score is the number of squares filled in correctly in the time limit.

The Five-Points Tests was used for evaluation of nonverbal executive functions [30]. This test requires production of novel designs under time constraints. The subject is given a sheet with a table containing five dots in each cell. The subject is asked to produce as many different figures or designs as possible in three minutes by connecting these five dots in each cell. 

The Wechsler Abbreviated Scale for Intelligence (WASI) was used for evaluation of general intellectual functioning [31]. 

Raw scores of all neuropsychological tests were transformed to age- and education-stratified standardized scores (SS for Digit Span and Digit Symbol Coding, IQ for WASI, and T-scores for other tests) based on available normative data. 

The Parkinson’s Disease Questionnaire (PDQ-39) was used to assess quality of life and symptom burden induced by PD [32]. The questionnaire contains 39 items that are summed in eight quality-of-life domains: Mobility, Activities of Daily Living, Emotional Well-Being, Stigma, Social Support, Cognitions, Communication and Bodily Discomfort. Domain scores are coded on a scale of 0 (perfect health as assessed by the measure) to 100 (worst health as assessed by the measure). A summary index score was also used for global evaluation of symptoms burden. 

### 2.3. Neurological Evaluation

Current analysis combines data from two different studies and two different rating scales of PD symptoms during each wave. During the first wave the Unified Parkinson’s Disease Rating Scale was used [33]. During the wave, two newly developed Parkinson’s Disease Composite Scales were included into the protocol [34]. Both scales overlap significantly; however, the UPDRS is much more comprehensive. In order to obtain comparable data about the presence of symptoms and their severity, we looked for equivalent items at both scales and developed a scoring system to evaluate the presence and severity of a symptom independently from the scale used. We intentionally excluded non-motor symptoms, such as emotional functioning, cognition and fatigue that are evaluated based on a patient’s self-report, because these symptoms are well represented in the PDQ-39. Each symptom was evaluated in a 5-point Likert type scale, where 0 represents absence of a symptom, 1—minimal, 2—mild, 3—moderate and 4 marked severe intensity of a symptom. More detailed information is provided in Supplement Table S2. 

### 2.4. Blood Sample Collection

Fasting venous blood samples (4 mL) were collected by vein puncture using coagulation-promoting tubes. The blood samples were left to stand up to 2 h at 4 °C and then processed for serum isolation at 1300× *g* for 10 min. The separated serum part was transferred into a 1.5 mL microcentrifuge tube and stored at −80 °C until miRNA isolation.

#### miRNA Isolation and cDNA Synthesis

EV isolation was carried out using commercially available kit “exoEasy Maxi/Midi Kit” (Cat. No. 76064), following the manufacturer ‘s instructions. Frozen serum samples were thawed at room temperature until they were completely liquid. EVs were extracted from 400 to 500 µL of the serum sample. After EV collection step, synthetic phosphorylated cel-miR-39-3p (Phos-UCACCGGGUGUAAAUCAGCUUG) (0.01 ng) was added to each sample to control and normalize the efficiency of RNA extraction. At the end, the samples were eluted in RNase-free water. The RNA yield was evaluated post-isolation by NanoDrop 2000. Ten ng of extracted microRNA was synthesized to cDNA with a “TaqMan Advanced miRNA cDNA Synthesis Kit” (Cat. A28007). After amplification and 10 times of dilution, cDNA was used for microRNA expression analysis.

### 2.5. miRNA Expression Analysis

Real-time PCR (RT-PCR) for miRNA expression was carried in three replicates using “TaqMan Fast Advanced Master Mix” (Thermo Fisher Scientific, Austin, TX, USA) and the following probes: hsa-miR-7-5p (Assay ID: 483061_mir), hsa-miR-21-5p (Assay ID: 477975_mir), hsa-miR-153-5p (Assay ID: 477922_mir), hsa-miR-155-5p (Assay ID: 483064_mir), hsa-miR-200a-3p (Assay ID: 478490_mir), and hsa-miR-214-5p (Assay ID: 477974_mir). In addition, cel-miR-39-3p (Assay ID: 478293_mir) was measured in order to normalize the data. RT-PCR was performer by the 7500 Fast Real-Time PCR system under the following conditions: 95 °C for 20 s followed by 45 cycles at 95 °C 3 s and 60 °C for 30 s. All reactions, including controls without samples, were examined in triplicates. The fold changes in miRNA expression were calculated using the Ct value of the normalizing control. All data were analyzed according to the comparative ∆Ct method.

### 2.6. Statistical Analysis

Statistical analysis was performed by Pearson’s correlation and Student’s *t* test using IMB SPSS statistics (version 27) and GraphPad Prism (version 7.0, Graph-Pad Software, San Diego, CA, USA) to evaluate the diagnostic importance of miRNAs expression levels between PD patient groups. The significance level of 0.05 (*p* < 0.05) was defined. 

## 3. Results

### 3.1. Patients’ Population and miRNA Expression

Blood serum samples of 95 PD patients were available for current analysis. Data of one patient was excluded due to very late onset of the illness (87 years), and one more patient was excluded due to very long duration of the illness (40 years). The final sample size consisted of 93 patients. Demographic and clinical characteristics of the samples are displayed in Table 1.

An initial panel of miRNAs was selected from the literature, suggested as potential molecular markers for PD. We aimed to examine whether α-synuclein-related serum EV miRNA’s (miR-7, miR-153, miR-214, miR-155, miR-21, miR-200a) were associated with patients’ gender, education or age. No reliable correlations were found between miRNA expression levels and the gender or education of the patients studied. Meanwhile, the detailed analysis between miRNA expression and PD patient age, illness duration and age at the onset of symptoms are shown in Table 2. Upregulation of miR-7, miR-155, miR-200a, and miR-214 was associated with the older age of patients, whereas upregulation of miR-21 was associated with younger PD patients and with the onset of symptoms at a younger age. Elevated miR-7 and miR-200a expression levels were associated with longer illness duration. 

### 3.2. Relationship between miRNA and Neurological Functioning

**Figure 1:**
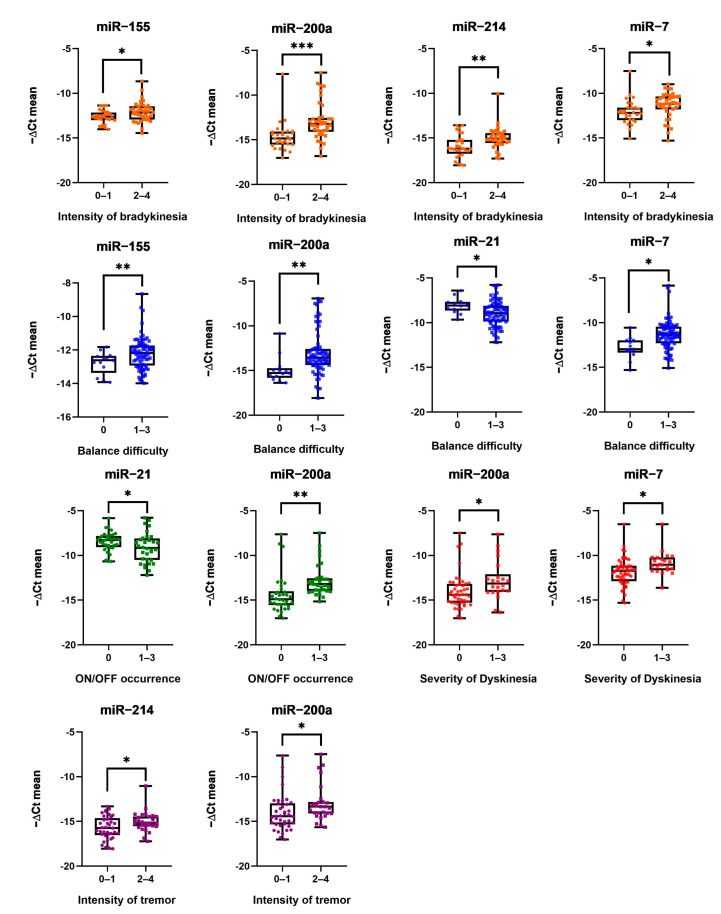
MiRNA expression and intensity of bradykinesia, balance difficulty, ON/OFF phenomena occurrence, severity of dyskinesia and intensity of tremor. Dots mark individual miRNA expression; whiskers mark minimum and maximum miRNA expression levels; and the black line in the middle marks the median of values. Numbers below mark patients grouped by intensity of symptoms from 0 to 4: 0—no symptoms, 1—low, 2—mild, 3—strong, and 4—severe. Expression of miRNA was normalized with cel-miR-39-3p; Student’s *t* test *: (*p* ≤ 0.05), **: (*p* ≤ 0.01), and ***: (*p* ≤ 0.001).

The relationships between serum EV miRNAs’ (miR-7, miR-21, miR-153, miR-155, miR-200a, miR-214) expression levels and patients’ neurological symptoms, such as bradykinesia, tremor, gait, balance, freezing, hallucinations or thoughts disorders (due to drug intoxication or dementia), dyskinesia, dystonia and motor fluctuations (ON/OFF) were studied. As shown in Table 3, Pearson’s correlation suggested significant associations between miR-7, miR-155, miR-200a and miR-214 upregulation and intensity of bradykinesia. MiR-200a and miR-214 expression was also upregulated as the severity of tremor became more intense. In addition, elevated miR-7, miR-155, miR-200a and downregulated miR-21 expression levels were related to impaired balance. Overexpression of miR-7 and miR-200a was also significantly associated with more frequent episodes of dyskinesia, whereas high expression of miR-214 and decreased levels of miR-21 correlated with more pronounced symptoms of the ON/OFF phenomena. After establishing statistically significant differences between miRNA expression and neurological functioning, it was decided to present them in more detail in Figure 1. Patients were divided into two groups according to the severity of symptoms and analyzed by using Student’s *t* test. None of the miRNAs tested were significantly associated with gait difficulty, freezing or hallucination symptoms.

### 3.3. Relationship between miRNA and Cognitive Functioning

In order to investigate whether miRNAs in blood serum EV’s could be suitable indicators to monitor cognitive functioning of patients with PD and to determine progression of the disease, it was decided to look for links between miRNA expression and an estimated general intellectual ability by measuring the verbal, nonverbal, and general cognition of individuals. Summary of data are presented in Table 4. Expression of miR-153 and miR-200a were found to be inversely related to verbal and general intellectual functioning in PD patients. PD patients with elevated expression of these miRNAs showed poorer intelligence assays using the Wechsler Abbreviated Scale for Intelligence. Due to involvement of the motor function as well as because of the bad general state of health, it was not always possible to perform complete psychological assessment. Several neuropsychological tests were used for more comprehensive assessment of PD patients’ cognitive functioning. A relationship analysis was conducted between miRNA expression levels and Trail Making Test parameters (TMT, Parts A and B) such as visual attention, psychomotor speed, mental flexibility and executive functioning. The results revealed that lower expressions of miR-155, miR-200a and miR-214 were associated with higher psychomotor speed. It has also been established that lower miR-200a expression is related to higher mental flexibility. No significant association was found between miRNA expression and phonemic fluency (letters), semantic fluency (animals), cumulative learning (RAVLT, A1-A5), delayed recall (RAVLT, A7) or recognition (RAVLT, recognition trial). Downregulated miR-153, miR-155, miR-200a and miR-214 expression levels were associated with higher non-verbal fluency. Also, lower miR-153 expression was associated with higher scores in attention span/working memory (WAIS-III, Digit span) and psychomotor speed with learning (WAIS-III, Digit Symbol Coding), as shown in Table 4.

### 3.4. Relationship between miRNA and Patient-Reported Health-Related Quality of Life

The PD Questionnaire (PDQ) was used to assess quality of life and symptom burden induced by PD. This questionnaire is summed in eight quality-of-life domains: Mobility, Activities of Daily Living (ADL), Emotional Well-Being, Stigma, Social Support, Cognitions, Communication and Bodily Discomfort. Domain scores are coded on a scale of 0 (perfect health) to 100 (worst health). A summary index score was also used for global evaluation of symptom burden. The correlations found between quality-of-life indicators and miRNA expression are summarized in Table 5. Few reliable correlations between miRNA expression and quality-of life–indicators have been identified. MiR-7, miR-21 and miR-200a expressions were related to the PD patient’s social support assessments. Mir-7 expression also was related to the PD patients’ emotional well-being. Although there was a lack of statistical reliability, there was a tendency for cognitive impairment in PD patients when miR-21 expression levels were increasing. 

**Figure 2:**
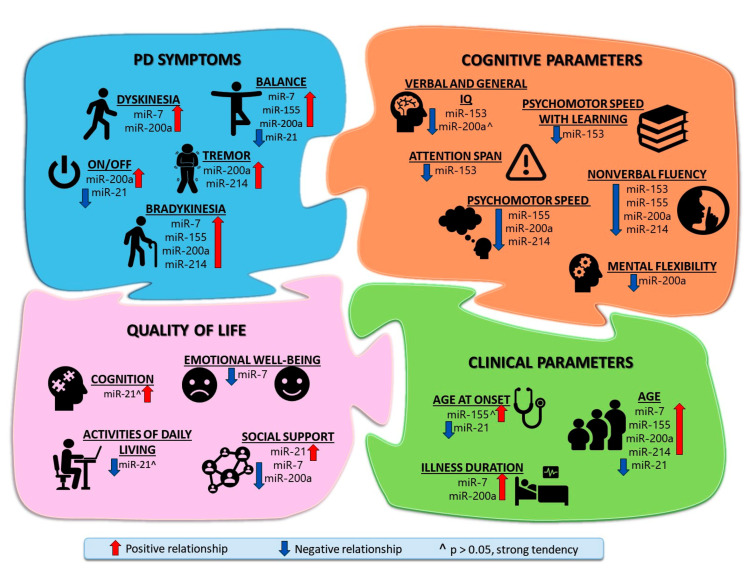
Relationship between Parkinson’s Disease symptoms and miRNAs expression in patients’ blood serum EVs. Comprehensive illustration of the modulatory role of miRNAs in impairment in PD shows the relationship (positive and negative dependence, and strong tendency) between miRNAs and clinical functions, PD symptoms, cognitive functions, and quality of life. PD symptoms and clinical parameters were assessed as they worsened, while quality of life and cognitive parameters were evaluated as they improved.

Summarizing all the obtained results, an illustration (Figure 2) was created, which shows with which parameters, which miRNAs were statistically reliably related or had strong trends.

## 4. Discussion

Extracellular miRNAs packed into EVs are protected from degradation and are therefore known for their stability as well as high sensitivity and specificity. These miRNA qualities could be used for PD diagnostics or innovative therapeutic approaches [35,36]. Despite the well described miRNA functions in PD, very little information is available about the levels of these miRNAs in serum EVs and their possible associations with PD motor and cognitive symptoms as well as quality-of-life evaluations. Our study revealed that both direct and indirect regulation of α-synuclein via miRNAs are equally important and significantly impact symptom severity, cognitive impairment, and quality of life in PD patients. Blood serum-derived EV miR-7, miR-153, miR-214, miR-155 and miR-200a expression levels are elevated and miR-21 is downregulated when motor and cognitive functions are declining in patients with PD. We also found that the duration of the illness and a lack of social support as well as low emotional well-being were associated with upregulation of miR-7 and miR-200a levels. An initial panel of miRNAs was selected according to their function in PD pathogenesis. Based on available publications, it is understood that miR-7 [37], miR-21 [21], miR-153 [38], miR-155 [20], miR-200a [39] and miR-214 [19] are involved in α-synuclein aggregation and neuronal death. However, previous research has showed that miRNA levels can be very dynamic and vary significantly depending on the tested sample type. Y. F. Yao et al. reported that miR-331 levels were barely expressed in plasma and were very abundant in exosomes, whereas miR-505 expression levels were only detectable in plasma but not exosomes for patients with PD [40]. Different testing samples provide different results to interpret. For example, miR-7 expression levels are upregulated in PD patients’ peripheral fluids compared to a healthy control (HC) when whole blood [41] or serum samples were tested; however, no significant differences were found when miR-7 exosomal expression was analyzed [42]. In contrast, animal and cell models have shown that miR-7 is decreased in dopaminergic neurons compared to a HC [43]. MiR-153 expression is downregulated in PD patients compared to a HC in plasma [44] and upregulated in CSF exosomes [45]; this could indicate a possible neuroprotective mechanism that tries to compensate for neuronal death. Studies using mouse brain as a model showed elevated miR-214 expression [46] as well as in plasma, serum, serum exosomes and CSF exosomes of patients with PD [47,48]. MiR-21 was upregulated in the midbrain of MPTP-treated mice compared to controls [49] and was downregulated in serum exosomes of PD patients compared to a HC [13]. MiR-155 expression is upregulated in EVs from PD patients’ blood plasma [50,51] and PBMC’s [52]. MiR-200a is overexpressed in SH-SY5Y cells following exposure to MPP+ [53]; however, N. Shakespear et al. reported decreased miR-200a expression from astrocyte-derived exosomes affected with MPP+ [54]. No information was found about miR-200a expression in PD patient samples. 

In conclusion, our study confirmed previous research that miR-153, miR-155 and miR-214 was upregulated and miR-21 downregulated in PD patients’ EVs, when comparing HCs to PD. Additionally, we provided a more detailed analysis of the correlation between these miRNAs and key features of PD patients, including motor and cognitive function decline, as well as quality-of-life parameters. Our study also complemented previous miR-200a research in PD cell models with new information about the serum EV miR-200a dynamic in disease progression.

## Figures and Tables

**Table 1:** Characteristics of patients with Parkinson’s disease.

Characteristic		N (%)
Gender	Male	41 (44.1%)
	Female	52 (55.9%)
Education	Without university degree	48 (51.6%)
	With university degree	45 (48.4%)
Type of treatment	Conservative	37 (39.8%)
	Deep Brain Stimulation	39 (41.9%)
	Gamma Knife Stereotactic Radiosurgery	11 (11.8%)
	Radio Frequency Ablation	6 (6.5%)
	Mean (SD)	Min.–Max.
Age at time of the assessment	62.14 (8.90)	39–82
Age at onset of first symptoms	53.21 (9.56)	29–75
Illness duration	8.93 (5.07)	1–20

**Table 2:** Relationship between miRNA and PD patients age and illness duration.

		miR-7	miR-21	miR-153	miR-155	miR-200a	miR-214
Age	Correlation Coefficient	0.214 *	−0.264 *	0.048	0.325 **	0.300 **	0.224 *
	Sig. (two-tailed)	0.04	0.011	0.652	0.002	0.004	0.033
	N	93	93	91	92	91	91
Illness duration	Correlation Coefficient	0.330 **	0.021	0.141	0.169	0.233 *	0.16
	Sig. (two-tailed)	0.001	0.839	0.183	0.107	0.027	0.129
	N	93	93	91	92	91	91
Age at symptoms onset	Correlation Coefficient	0.019	−0.266 **	−0.027	0.199	0.125	0.141
	Sig. (two-tailed)	0.857	0.01	0.797	0.057	0.238	0.183
	N	93	93	91	92	91	91

** Correlation is significant at the 0.01 level (two-tailed). * Correlation is significant at the 0.05 level (two-tailed).

**Table 3:** Relationship between miRNA and neurological functioning.

		miR-7	miR-21	miR-153	miR-155	miR-200a	miR-214
Bradykinesia	Pearson’s r	0.271 *	−0.195	0.109	0.333 **	0.382 **	0.447 **
	Sig. (two-tailed)	0.028	0.117	0.392	0.006	0.002	0.000
	N	66	66	64	66	65	64
Tremor	Pearson’s r	0.194	−0.204	−0.081	0.18	0.291 *	0.285 *
	Sig. (two-tailed)	0.118	0.1	0.527	0.149	0.019	0.023
	N	66	66	64	66	65	64
Gait	Pearson’s r	0.213	0.207	−0.081	0.07	0.043	0.139
	Sig. (two-tailed)	0.086	0.095	0.525	0.576	0.735	0.273
	N	66	66	64	66	65	64
Balance	Pearson’s r	0.474 **	−0.244 *	0.049	0.248 *	0.334 **	0.234
	Sig. (two-tailed)	0	0.048	0.7	0.044	0.007	0.062
	N	66	66	64	66	65	64
Freezing	Pearson’s r	0.173	−0.112	−0.041	0.05	0.017	0.084
	Sig. (two-tailed)	0.164	0.37	0.748	0.692	0.895	0.51
	N	66	66	64	66	65	64
Hallucinations	Pearson’s r	0.065	−0.175	−0.075	−0.062	−0.029	0.107
	Sig. (two-tailed)	0.608	0.166	0.565	0.627	0.824	0.409
	N	64	64	62	64	63	62
Dyskinesia	Pearson’s r	0.252 *	0.089	0.098	0.11	0.259 *	−0.092
	Sig. (two-tailed)	0.041	0.476	0.443	0.378	0.037	0.471
	N	66	66	64	66	65	64
ON/OFF	Pearson’s r	0.123	−0.340 **	0.012	0.03	0.226	0.253 *
	Sig. (two-tailed)	0.324	0.005	0.924	0.809	0.07	0.044
	N	66	66	64	66	65	64

** Correlation is significant at the 0.01 level (two-tailed). * Correlation is significant at the 0.05 level (two-tailed).

**Table 4:** Relationship between miRNA and cognitive functioning.

		miR-7	miR-21	miR-153	miR-155	miR-200a	miR-214
WASI Verbal IQ	Pearson’s r	−0.175	−0.056	−0.434 **	−0.159	−0.304	0.019
	Sig. (two-tailed)	0.286	0.733	0.006	0.334	0.06	0.909
	N	39	39	38	39	39	38
WASI Nonverbal IQ	Pearson’s r	−0.055	−0.138	−0.255	−0.135	−0.234	0.157
	Sig. (two-tailed)	0.756	0.436	0.153	0.445	0.182	0.382
	N	34	34	33	34	34	33
WASI General IQ	Pearson’s r	−0.22	−0.092	−0.433 **	−0.227	−0.308	0.144
	Sig. (two-tailed)	0.198	0.592	0.009	0.182	0.067	0.409
	N	36	36	35	36	36	35
Psychomotor speed (Trail making, Part A)	Pearson’s r	−0.19	−0.113	−0.222	−0.289 *	−0.297 *	−0.277 *
	Sig. (two-tailed)	0.105	0.339	0.061	0.013	0.011	0.019
	N	74	74	72	73	73	72
Mental flexibility (Trail Making, Part B)	Pearson’s r	−0.183	0.031	−0.218	−0.212	−0.270 *	−0.155
	Sig. (two-tailed)	0.121	0.796	0.068	0.073	0.022	0.196
	N	73	73	71	72	72	71
Non-verbal fluency	Pearson’s r	−0.222	−0.082	−0.291 *	−0.302 *	−0.271 *	−0.384 **
	Sig. (two-tailed)	0.064	0.499	0.016	0.012	0.025	0.001
	N	70	70	68	69	69	68
Attention span/working memory (WAIS-III, Digit span)	Pearson’s r	−0.013	−0.049	−0.216 *	0.047	−0.039	0.092
	Sig. (two-tailed)	0.903	0.656	0.05	0.669	0.727	0.407
	N	85	85	83	84	84	83
Psychomotor speed with learning (WAIS-III, Digit Symbol Coding)	Pearson’s r	−0.129	−0.169	−0.305 *	−0.135	−0.079	−0.039
	Sig. (two-tailed)	0.308	0.182	0.015	0.289	0.538	0.759
	N	64	64	63	64	63	63

** Correlation is significant at the 0.01 level (two-tailed). * Correlation is significant at the 0.05 level (two-tailed).

**Table 5:** Relationship between miRNA and patient-reported health-related quality of life.

		miR-7	miR-21	miR-153	miR-155	miR-200a	miR-214
PDQ_Mobility	Pearson’s r	0.117	−0.148	−0.121	−0.062	0.124	−0.037
	Sig. (two-tailed)	0.301	0.189	0.292	0.589	0.276	0.746
	N	80	80	78	79	79	78
PDQ_ADL	Pearson’s r	0.159	−0.214	−0.09	−0.067	0.068	−0.002
	Sig. (two-tailed)	0.16	0.057	0.433	0.559	0.55	0.985
	N	80	80	78	79	79	78
PDQ_Emotional	Pearson’s r	−0.298 **	0.09	−0.196	−0.1	−0.104	−0.132
	Sig. (two-tailed)	0.007	0.428	0.086	0.378	0.363	0.251
	N	80	80	78	79	79	78
PDQ_Stigma	Pearson’s r	−0.153	0.043	−0.086	−0.064	−0.096	−0.071
	Sig. (two-tailed)	0.176	0.706	0.452	0.574	0.398	0.536
	N	80	80	78	79	79	78
PDQ_Social_support	Pearson’s r	−0.343 **	0.299 **	−0.221	−0.101	−0.236 *	−0.044
	Sig. (two-tailed)	0.002	0.007	0.051	0.377	0.036	0.705
	N	80	80	78	79	79	78
PDQ_Cognition	Pearson’s r	−0.137	0.212	−0.047	0.098	−0.083	0.002
	Sig. (two-tailed)	0.224	0.059	0.681	0.39	0.465	0.989
	N	80	80	78	79	79	78
PDQ_Communication	Pearson’s r	−0.106	0.12	−0.091	−0.107	−0.089	−0.18
	Sig. (two-tailed)	0.349	0.289	0.43	0.347	0.433	0.115
	N	80	80	78	79	79	78
PDQ_Bodily_discomfort	Pearson’s r	−0.026	−0.197	−0.024	0.064	0.155	0.024
	Sig. (two-tailed)	0.82	0.08	0.834	0.578	0.174	0.835
	N	80	80	78	79	79	78
PDQ39_Summary_Index	Pearson’s r	−0.146	−0.017	−0.193	−0.089	−0.018	−0.086
	Sig. (two-tailed)	0.196	0.883	0.091	0.434	0.875	0.453
	N	80	80	78	79	79	78

** Correlation is significant at the 0.01 level (two-tailed). * Correlation is significant at the 0.05 level (two-tailed).

## Data Availability

Data related to this research are available upon individual request.

## Supplementary Materials

The following supporting information can be downloaded at: https://www.mdpi.com/article/10.3390/biom14081000/s1, Table S1: Descriptive statistics for neuropsychological tests; Table S2: Scoring of symptoms severity based in items of the Parkinson’s Disease Composite Scale and the Unified Parkinson’s Disease Rating Scale.
