# Supplementary material for: Association of Serum Extracellular Vesicle miRNAs with Cognitive Functioning and Quality of Life in Parkinson’s Disease
Source: Biomolecules. 2024 Aug 13;14(8):1000. doi: 10.3390/biom14081000 (PMC11352584; doi:10.3390/biom14081000)
Supplement: Supplementary file 1 [file biomolecules-14-01000-s001.zip › biomolecules-3122251-supplementary.pdf]

SUPPLEMENTARY DATA

Supplement Table S1. Descriptive statistics for neuropsychological tests.

Psychological function (test)	N	Minimum	Maximum	Mean	Std. Deviation
WASI Verbal IQ	39	68	141	112,03	15,37
WASI Nonverbal IQ	34	81	131	100,76	12,10
WASI General IQ	36	76	141	108,17	13,12
Psychomotor speed (Trail making, Part A)	74	0	62	35,09	17,56
Mental flexibility (Trail Making, Par B)	73	0	66	27,96	21,25
Phonemic fluency (Letters)	85	31	67	47,24	8,68
Semantic fluency (Animals)	86	30	63	46,88	7,06
Cumulative learning (RAVLT, A1-A5)	84	19	62	41,04	9,83
Delayed recall (RAVLT, A7)	82	19	63	42,69	9,65
Recognition (RAVLT, recognition trial)	82	0	57	23,76	19,25
Non-verbal fluency	70	18	61	39,98	9,64
Attention span / working memory (WAIS-III, Digit span)	85	3	15	9,85	2,14
Psychomotor speed with learning (WAIS-III, Digit Symbol Coding)	64	5	18	10,30	2,75
Health related quality of life (The PDQ-39)	80	7	63	38,05	12,73

Supplement Table S2. Scoring of symptoms severity based in items of the Parkinson's Disease Composite Scale and the Unified Parkinson's Disease Rating Scale.

Symptom	The Parkinson's Disease Composite Scale	Equivalent item at the Unified Parkinson's Disease Rating Scale
Bradykinesia	Item 1: Bradykinesia (Total of finger tapping, alternating hand movements, legs agility, total impression of body bradykinesia)	Item 3.14 Global spontaneity of movement (body bradykinesia). This global rating combines all observations on slowness, hesitancy and small amplitude and poverty of movement in general, including a reduction of gesturing, and of crossing the leg.
Tremor	Item 2: Tremor (Total four limbs)	Item 3.18. Constancy of rest tremor. This item receives one rating for all rest tremor and focuses on the constancy of the rest tremor during the examination period when different body parts are variously at rest.
Gait	Item 3: Gait	Item 3.10. Gait
Balance/Postural stability	Item 4: Balance/postural stability	Item 3.12 Postural stability
Freezing	Item 5: Freezing	Item 3.11. Freezing of gait
Hallucinations or thought disorder (due to drug intoxication or dementia)	Item 12: Hallucinations or thought disorder (due to drug intoxication or dementia)	Item 1.2. Hallucinations and psychosis
Dyskinesia	Item 13: Dyskinesia (or if present, Rate the severity of camptocormia and/or Pisa syndrome)	Item 4.1. Time spent with dyskinesias
Dystonia	Item 14: Dystonia	Item 4.6: Painful off-state dystonia
ON/OFF	Item 15: ON/OFF	Item 4.3: Time spent in the off state
